# Network science meets algebraic topology

**DOI:** 10.1093/nsr/nwz066

**Published:** 2019-05-28

**Authors:** Lingqing Shen, David M Walker, Michael Small

**Affiliations:** 1 Department of Mathematics, Nanjing University, China; 2 Department of Mathematics & Statistics, University of Western Australia, Australia; 3 Mineral Resources, Commonwealth Scientific and Industrial Research Organisation, Australia

Network science is largely consumed with the study of graphs with heterogeneous degree: typically large graphs with no strong topological symmetry. Network science borrows approaches from statistical physics to understand this heterogeneous structure, in a manner quite distinct from the rich symmetry of the graphs that are studied by algebraic graph theorists. Shi *et al.* [1] move toward bridging this divide by switching the focus from the analysis of node degree sequence to cycles. Rather than focusing on a statistical description of degrees and the connective paths between nodes, they instead study cycles of specific order within networks.

**Figure 1.**
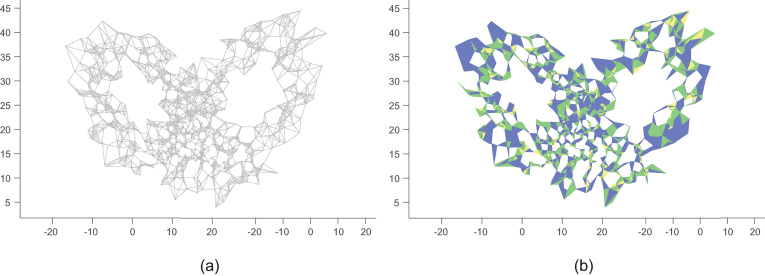
(a) Following [2], a network constructed from a Markov transition matrix of a quantization of the Lorenz system. (b) Simplicial complex induced from the network in (a). The 2-simplexes are marked by blue triangles, 3-simplexes by green tetrahedrons and 4-simplexes by yellow. Persistent homology computed from this complex reveals signatures unique to deterministic chaotic dynamics.

Identifying cycles in a network is a deceptively difficult problem, and has long been a mainstay of computational network science. While it is obvious what we mean by a cycle, it is much harder to exhaustively enumerate the cycles of a large network. In 2008, Xu *et al.* [2] presented the first of what has become a modest industry of techniques aimed at uncovering dynamical properties of deterministic dynamical systems by representing time series as complex networks. The networks produced were particularly evocative as they contained many holes and loops—much like a work of crochet (see Fig. 1a). At the time, the only computationally feasible approach to study these was to examine the motif super-family: the relative frequency of distinct connected (but not fully connected) subgraphs of fixed size. Shi *et al.* [1] describe a broader framework that shows great promise for the understanding of networks such as these, as well as networks arising in a wide range of other physical and engineering settings.

The key idea is to borrow the rich mathematical structure of algebraic topology as it applies to graph theory—to compute characteristic number, homology group, Betti numbers and persistent homology [3]. Collectively, these methods quantify the local dimension of solid objects by comparing volume and boundary. When applied to networks, this means decomposing a network to its fully connected sub-components (the ‘simplexes’ of [1]) and their boundaries. Figure 1 shows our application of this simplicial approach to a network constructed from the time series of the Lorenz system [4]. The distribution, interconnection and placement of simplexes within this simplicial complex reveal structural properties of the underlying chaotic dynamical system that we are only just beginning to understand.

This leads to a new framework of *clique vector spaces* by decomposing the graph into cycles, simplexes and boundaries. One can then obtain technical, but also fairly easily computable, definitions for chain group, cycle group, boundary group and homology group, all of which can be obtained directly from the network.

In their seminal description of small-world networks, Watts and Strogatz [5] show that regular networks with a small number of long-range connections gain both the strong clustering of a lattice and the small diameter (or short path-length) of random graphs. They argue that this combination is particularly useful for synchronization of dynamics on a network. In [1], the new paradigm of network homology is applied to show that characteristic number is indicative of network synchronizability. Moreover, simulations of disease transmission on networks indicate that regular network topology—and in particular the exemplar totally homogeneous network—is most effective for transmission. In the future we can expect methods like those introduced in [1] to have a significant impact on our understanding of structure in large complicated networks. This is particularly important for network models of real systems with structure much more complicated than described by the statistical properties of degree sequence [6].
